# Enhancing prime editing by fusing polymerase substrate-binding proteins to reverse transcriptase

**DOI:** 10.1093/nar/gkag657

**Published:** 2026-06-27

**Authors:** Dongdong Zhao, Ting Wang, Lu Zhang, Taixin Sha, Xiumei Zhao, Yanfang Lu, Rongfei Wang, Tao Fu, Jikai Liu, Yanrong Li, Dan Zhao, Wenzhu Tang, Changhao Bi, Xueli Zhang

**Affiliations:** Tianjin Institute of Industrial Biotechnology, Chinese Academy of Sciences, Tianjin 300308, China; University of Chinese Academy of Sciences, Beijing 100049, China; Key Laboratory of Engineering Biology for Low-Carbon Manufacturing, Tianjin 300308, China; Tianjin Institute of Industrial Biotechnology, Chinese Academy of Sciences, Tianjin 300308, China; University of Chinese Academy of Sciences, Beijing 100049, China; Tianjin Institute of Industrial Biotechnology, Chinese Academy of Sciences, Tianjin 300308, China; School of Biological Engineering, Dalian Polytechnic University, Dalian 116034, China; Tianjin Institute of Industrial Biotechnology, Chinese Academy of Sciences, Tianjin 300308, China; School of Biological Engineering, Dalian Polytechnic University, Dalian 116034, China; Tianjin Institute of Industrial Biotechnology, Chinese Academy of Sciences, Tianjin 300308, China; Tianjin Institute of Industrial Biotechnology, Chinese Academy of Sciences, Tianjin 300308, China; Tianjin Institute of Industrial Biotechnology, Chinese Academy of Sciences, Tianjin 300308, China; Tianjin Institute of Industrial Biotechnology, Chinese Academy of Sciences, Tianjin 300308, China; Tianjin Institute of Industrial Biotechnology, Chinese Academy of Sciences, Tianjin 300308, China; Tianjin Institute of Industrial Biotechnology, Chinese Academy of Sciences, Tianjin 300308, China; Tianjin Institute of Industrial Biotechnology, Chinese Academy of Sciences, Tianjin 300308, China; School of Biological Engineering, Dalian Polytechnic University, Dalian 116034, China; Tianjin Institute of Industrial Biotechnology, Chinese Academy of Sciences, Tianjin 300308, China; University of Chinese Academy of Sciences, Beijing 100049, China; Key Laboratory of Engineering Biology for Low-Carbon Manufacturing, Tianjin 300308, China; Tianjin Institute of Industrial Biotechnology, Chinese Academy of Sciences, Tianjin 300308, China; University of Chinese Academy of Sciences, Beijing 100049, China; Key Laboratory of Engineering Biology for Low-Carbon Manufacturing, Tianjin 300308, China

## Abstract

Prime editing (PE) enables precise small DNA changes yet often shows modest efficiency in human cells. Recent advances indicate that the activity of the reverse transcriptase (RT) component is a key determinant of PE performance. Here, we adapt a principle from natural polymerases by fusing a polymerase substrate binding protein (PSBP) with RT to enhance PE. In a twelve-member screen, multiple PSBPs increased editing efficiency, with a compact HMG box module, NHP6A, emerging as a representative lead fusion. NHP6A fused to RT significantly increases intended edits across substitutions, insertions, and deletions while indels remain low. The enhancement is broadly compatible with a nicking sgRNA (PE3), transient mismatch repair inhibition via MLH1dn (PE4), and structured 3′ pegRNA extensions (epegRNA). The PSBP module also synergizes with the La protein-assisted pegRNA tail stabilization (the PE7 system) for additional improvements. Using NHP6A together with La, we efficiently installed four clinically relevant alleles with high product purity. Finally, we validate that PSBP fusion can be consolidated into a single component prime editor without loss of activity. These results establish PSBP fusion as a precise route to improve prime editing outcomes and support integrating compact accessory modules into next-generation PE platforms.

## Graphical Abstract

**Graphical Abstract:**
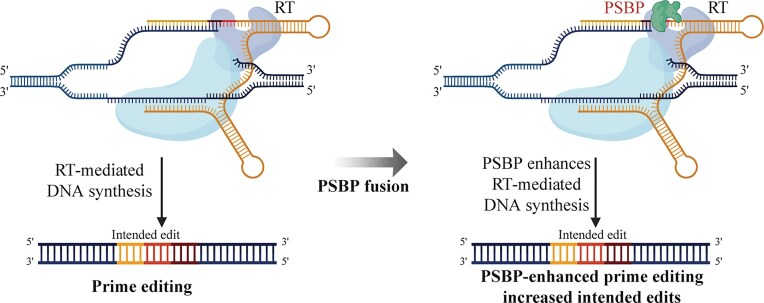

## Introduction

Prime editing (PE) couples a Cas9 (H840A) nickase to a reverse transcriptase (RT) and a pegRNA that both targets the genomic site and encodes the desired change, enabling precise substitutions, short insertions, and deletions without a donor template [1, 2]. In principle, PE could correct a large fraction of pathogenic variants, yet in human cells its efficacy is often constrained by modest editing yields and byproduct formation [3]. The multistep reaction is inherently inefficient: after pegRNA-guided nicking and hybridization of the pegRNA 3′ extension to the nicked strand, the RT must copy the RNA template into DNA [4]. Incomplete extension or premature resolution can lower productive editing and generate mixed heteroduplexes that cellular repair may resolve unfavorably. These limitations have motivated several complementary engineering strategies.

Recent advances in prime editing engineering have focused on evolving the RT enzyme [5–7], stabilizing the pegRNA [8], and modulating mismatch repair (MMR) to counteract these limitations [4, 9]. For example, the PEmax combines codon optimization, strengthened nuclear localization, and expression refinements, which together yield consistently higher editing across diverse contexts. Directed evolution of the RT produced PE6 variants with further activity gains [5]. In parallel, pegRNA stabilization strategies have proven effective. Attaching structured RNA motifs to pegRNA 3′ ends (epegRNAs) increases editing efficiencies without detectably elevating off-target editing [8], and introducing strategic mismatches in the pegRNA protospacer (mpegRNAs) can reduce pegRNA secondary structure and prolong engagement between the editor and the substrate [10]. Another powerful approach is suppression of mismatch repair [4]. Transient inhibition of this pathway, which otherwise recognizes and reverses heteroduplex edits, improves both editing frequency and product purity, and coexpression of a dominant negative MLH1 (MLH1dn, also referred to as PE4) is a representative implementation. More recently, researchers enhanced prime editing by fusing RNA-interacting domains to the editor. In particular, the PE7 system appends the human La domain to engage a poly (U) pegRNA tail, stabilizing the pegRNA and RT complex and yielding pronounced gains across multiple contexts [11]. Together, these advances show that enzyme, RNA template, and repair levers can each relieve distinct bottlenecks in prime editing, yet opportunities remain to improve efficiency without sacrificing precision by targeting additional facets of the mechanism.

A widely used principle in nucleic acid synthesis is recruitment of polymerase accessory factors that clasp or stabilize the primer-template duplex to sustain synthesis under impediments. Such polymerase substrate binding proteins (PSBPs) have been implemented in diverse ways across polymerase families. For thermostable DNA polymerases, appending compact duplex-engagement modules [e.g. helix-hairpin-helix (HhH) repeats from topoisomerase V] increases thermostability and supports efficient primer extension at elevated temperatures by maintaining processive synthesis without compromising catalytic rate [12]. In φ29 systems, fusing short HhH segments adjacent to the product-exit path enhances DNA association and improves isothermal amplification performance while retaining normal elongation kinetics, consistent with a substrate-engagement mechanism rather than altered catalysis. More generally, tethering compact duplex-engagement modules, such as Sso7d-like folds, to DNA polymerases increases processivity and enhances tolerance to inhibitors [13].

**Figure 1.**
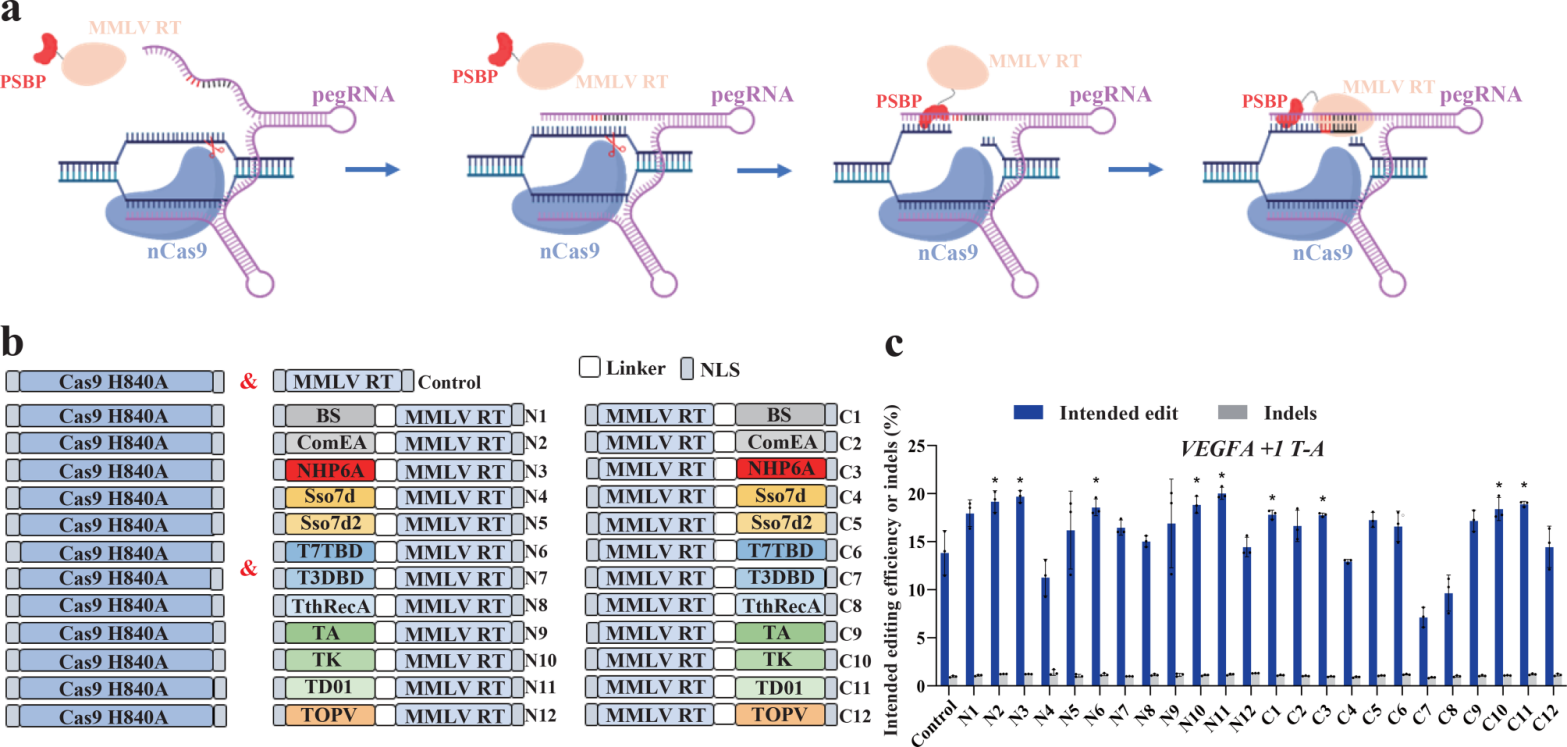
Overview of PSBP-augmented prime editors and initial screen of candidate fusions. (**a**) Schematic of the prime editing mechanism with a polymerase substrate binding protein (PSBP) fused to the MMLV RT to help stabilize the primer-template hybrid (the pegRNA 3′ extension annealed to target DNA) during reverse transcription. (**b**) Twelve candidate PSBPs were fused to the RT in either N- or C-terminal orientations (N1 to N12 and C1 to C12). These candidates include a yeast HMG-box protein (NHP6A), archaeal DNA-binding proteins (Sso7d and Sso7d2), phage T7 and T3 DNA polymerase thioredoxin-binding domains (T7TBD and T3TBD), Thermus thermophilus RecA (TthRecA), a Topo V HhH domain (TOPV), and other basic DNA-binding proteins from bacteria (“ComEA” and Bacillus spurious HU, labeled BS) or archaea (TA and TK), as well as a synthetic DNA-binding domain (TD01). (**c**) Prime editing outcomes at the VEGFA + 1 T-to-A site for each RT–PSBP fusion tested in the split PE system. Blue bars indicate intended edit percentage and gray bars indicate indel percentage (mean ± SD, *n* = 3); dots represent individual values from three independent biological replicates. Asterisks indicate statistically significant increases in intended editing compared with the RT-only control, determined by two-tailed unpaired Student’s *t*-tests (*P < .05).

Importantly, the same concept extends to reverse transcription. Attaching a small substrate-engagement module related to Sso7d to Moloney murine leukemia virus RT increased processive cDNA synthesis and improved tolerance to common reaction inhibitors, illustrating that compact PSBPs can fortify an RT-primer-template complex without impairing turnover [14]. Building on this polymerase-agnostic principle, we hypothesized that fusing analogous PSBPs to the PE RT would stabilize the nascent RNA–DNA heteroduplex during extension, prolong RT dwell time, and better support strand displacement in the presence of local structural obstacles, thereby converting more initiation events into productive prime editing outcomes (Fig. 1a).

To test this hypothesis, we engineered a series of prime editor variants in which distinct PSBPs were positioned on the reverse transcriptase scaffold and evaluated their performance in human cells. Most PSBPs increased editing efficiency. The leading design, an NHP6A-RT fusion that we term N6RT, raised the proportion of intended products across substitutions, insertions, and deletions while indel formation remained low. The strategy was compatible with and additive to established enhancements, including nicking in PE3, transient mismatch repair inhibition with MLH1dn in PE4, engineered pegRNAs such as epegRNAs, and La-assisted pegRNA tail stabilization in a PE7-related configuration. Taken together, these findings establish PSBP fusion as a general and precise means to improve prime editing efficiency, likely by supporting RT-associated substrate engagement during prime editing.

## Materials and methods

### Strains and culture conditions

For routine cloning, *E. coli* DH5α was grown at 37°C in Luria–Bertani (LB) broth (1% w/v tryptone, 0.5% w/v yeast extract, 1% w/v NaCl) or on LB agar (LB + 1.5% agar). Where appropriate, ampicillin was used at 100 µg ml⁻¹. Cultures were shaken at 220 rpm. Plates were incubated 12–16 h.

### Plasmid construction

PE2 plasmid was obtained from Addgene (#132775). Prime editor variants were assembled by Golden Gate cloning. Candidate PSBP genes or domains and oligonucleotides were synthesized by GENEWIZ. Candidate PSBP genes or domains were inserted at specified locations in the PE2 or split-PE backbone. For PSBP-RT fusion constructs, PSBPs were fused to RT through the amino acid linker SGGSGGSGGS. For split editors, the nCas9 and RT, with or without PSBP fusion, were expressed on separate plasmids with matching nuclear localization sequences. The La protein was cloned into a plasmid with a CMV promoter for co-transfection in La experiments. To construct pegRNA expression plasmids, a U6-driven gRNA cloning backbone was used [10]. Inserts containing the spacer, RTT, and PBS sequences were generated by PCR using primers encoding the corresponding elements and cloned into the pegRNA backbone. All constructs were verified by Sanger sequencing. The PSBP sequences are provided in Supplementary Sequences 1. The protospacer and 3′ extension sequences of pegRNAs/epegRNAs and nicking sgRNA spacers are provided in Supplementary Table S1.

### Cell culture and transfection

HEK293T, HepG2, and HeLa cells (ATCC) were cultured in Dulbecco’s minimal essential medium (DMEM, Gibco), supplemented with 10% (vol/vol) fetal bovine serum (FBS) and 1× penicillin streptomycin (Corning). Cells were incubated and cultured at 37°C with 5% CO_2_. For editing experiments, cells were seeded in 48-well plates and transfected at 40%–50% confluency using PEI (Yeasen) or Lipofectamine 2000 (Thermo Scientific). A typical transfection included 400 ng editor plasmid (for split, 200 ng each of nCas9 and RT plasmid), 125 ng pegRNA plasmid, 50 ng nicking sgRNA plasmid (if PE3), and 100 ng MLH1dn or La plasmid (if applicable). Puromycin (Merck, 4 μg/ml) was added ~24 h post-transfection to enrich for transfected cells and maintained for 4 days, after which cells were harvested for analysis.

### High-throughput sequencing and analysis

Cells cultured in 48-well plates (∼1.5 × 10^5^ cells per well) were washed with PBS and lysed in 30 μl QuickExtract DNA Extraction Solution (Epicentre) supplemented with 0.3 μl Proteinase K (Solarbio, 10 mg/ml). The lysates were incubated at 55°C for 10 min and 80°C for 3 min. For each PCR reaction, 2 μl of cell lysate, corresponding to ~1 × 10^4^ cell equivalents, was used as the template. Target regions were amplified using 2× EasyTaq PCR SuperMix (OKOID). PCR was performed using the following conditions: 95°C for 5 min, 30 cycles of 95°C for 30 s, 55°C for 30 s, and 72°C for 40 s, followed by 72°C for 10 min. Amplicons were pooled and sequenced on an Illumina MiniSeq platform using 2 × 150 bp paired-end sequencing. Sequencing reads were delivered as demultiplexed FASTQ files after base calling, quality filtering, and index-based sample separation.

Demultiplexed reads were analyzed using CRISPResso2 [15]. The original genomic sequences and intended edited sequences are provided in Supplementary Table S2, and the deep-sequencing PCR primers are provided in Supplementary Table S3. Editing efficiency was calculated as the percentage of aligned reads containing the intended edit among the total aligned reads. Indel frequency was calculated as the percentage of aligned reads containing insertions or deletions at the analyzed locus. For PE3 experiments, indels at the corresponding nicking sgRNA region were also assessed where applicable. Data were plotted using GraphPad Prism 9.

### Western blot analysis

HEK293T cells were collected 24 h after transfection and lysed in cell lysis buffer (Beyotime). Proteins were separated by sodium dodecyl sulfate–polyacrylamide gel electrophoresis and transferred to NC membranes. HA-tagged RT and N6RT were detected using an anti-HA antibody (Proteintech), and β-actin was used as the loading control.

### Cell morphology, proliferation, and apoptosis analysis

HEK293T cells were transfected with RT or N6RT plasmids under the same conditions used for editing experiments. Cell morphology was recorded by bright-field microscopy at 0, 24, and 48 h after transfection. Cell proliferation was measured using Cell Counting Kit-8 (Yeasen) at 0, 24, 48, and 72 h after transfection. Briefly, CCK-8 reagent was added to each well, and cells were incubated at 37°C before absorbance was measured at 450 nm. For apoptosis analysis, cells were collected after transfection and stained using an Annexin V-FITC/PI apoptosis detection kit (MULTI SCIENCES) according to the manufacturer’s instructions. Stained cells were analyzed by flow cytometry, and the percentages of cells in each quadrant were calculated.

### Statistical analysis

All the data were from three independent cell cultures. Data are presented as mean ± SD. For the initial PSBP-fusion screen, each PSBP-fusion condition was compared with the RT-only control using two-tailed unpaired Student’s *t*-tests. For pooled comparisons across matched target sites or edit classes, statistical significance was evaluated using two-tailed paired t-tests. Statistical analyses were performed using GraphPad Prism 9. *P* < .05 was considered to be statistically significant.

## Results

### Screening candidate PSBP-RT fusions

Guided by how natural DNA and RNA polymerases operate with accessory modules, we assembled a 12-member panel of polymerase substrate-binding proteins spanning bacterial, archaeal, and eukaryotic organisms (Fig. 1b) that are known or proposed to enhance primer-template engagement or polymerase performance. These included a compact HMGB family module, NHP6A [16]; archaeal chromatin proteins Sso7d and Sso7d2, commonly used to increase polymerase processivity [13, 14]; thioredoxin-binding domains from phage polymerases (T7TBD and T3TBD) [17, 18] that recruit host thioredoxin to raise processivity; TthRecA [19], which stabilizes and remodels DNA structures and improves amplification outcomes; an HhH domain from topoisomerase V (TOPV) [12, 20], implicated in improved processivity and salt tolerance when fused to polymerases; and additional basic or nucleic acid-engaging prototypes (ComEA, BS, TA, TK, TD01) [21–25] included to diversify duplex-engagement strategies.

A recent study showed that nCas9 and MMLV RT, when expressed separately, perform as efficiently as intact PE2 in human cells [7]. To specifically assess PSBP-driven enhancement of RT activity independent of Cas9, we therefore used the split PE system in which nCas9 and RT are delivered as independent components, and candidate PSBPs are fused only to the RT (N- or C-terminal to RT). At a standard VEGFA + 1 T-to-A editing site, statistical analysis showed that nine PSBP-fusion constructs significantly increased intended editing relative to the RT-only control in the split PE system (Fig. 1c). These significant constructs corresponded to six PSBPs, with significant effects observed for either N-terminal or C-terminal RT fusions depending on the PSBP. Most PSBP fusions maintained comparably low indel rates, indicating that increased intended editing did not generally come at the cost of higher indel formation.

Among the active candidates, we selected NHP6A-RT, termed N6RT, for further characterization. NHP6A represents a compact HMG-box protein with established nucleic-acid-binding activity, making it a suitable module to evaluate the PSBP-fusion strategy in detail. The activity of multiple PSBPs, including TK and TD01, further supports the broader potential of PSBP fusion for improving prime editing.

### N6RT Elevates editing efficiency across diverse loci while preserving specificity

**Figure 2.**
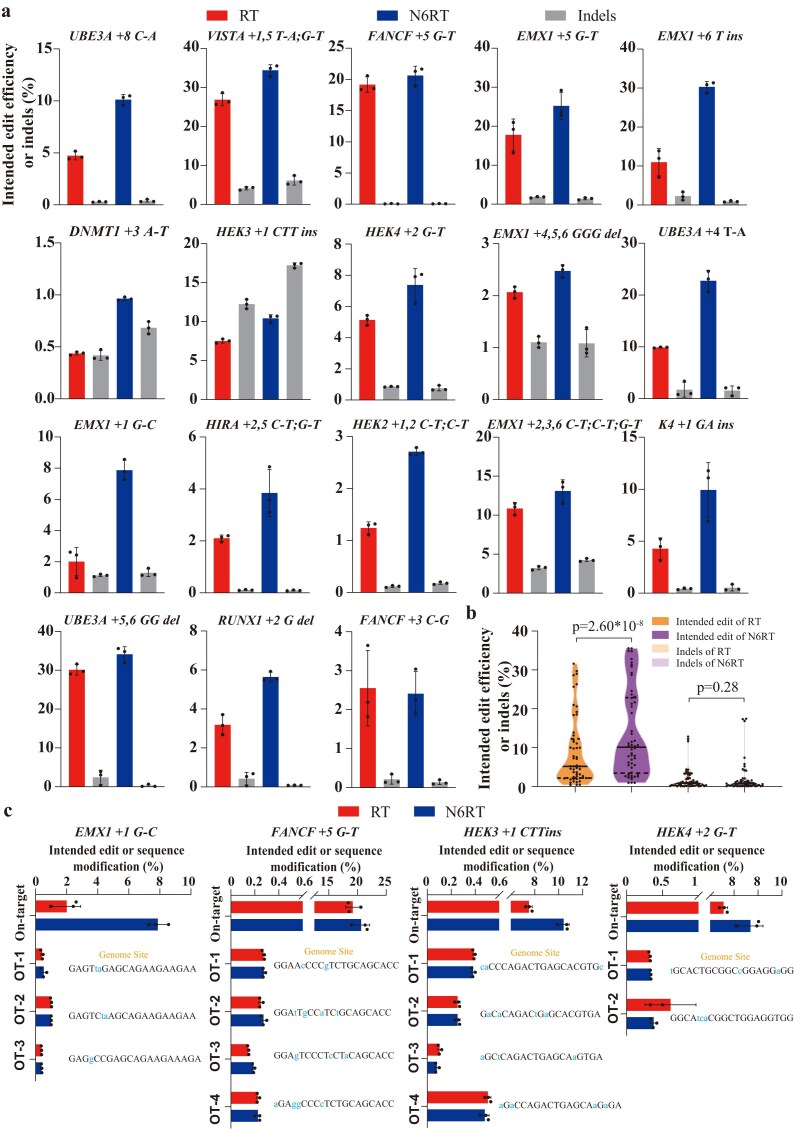
Performance of the N6RT fusion editor across multiple targets. (**a**) Editing outcomes at various endogenous loci in HEK293T cells comparing the standard prime editor to the N6RT fusion editor. For each target, the percentage of sequencing reads with the intended edit is higher with N6RT, while indel percentages remain similarly low for both editors. Nucleotide positions are numbered relative to the Cas9 nick site, with the first nucleotide after the nick defined as position +1. (**b**) Pooled analysis of editing efficiencies and indel rates across all tested loci for RT versus N6RT. N6RT significantly increases the fraction of intended edits (paired t-test, *P* = 2.6 × 10⁻⁸), whereas indel rates are not significantly different between RT and N6RT (paired t-test, *P* = .28). (**c**) Candidate off-target analysis for representative targets, including EMX1, FANCF, HEK3, and HEK4. Products corresponding to the designed on-target intended edits were not detected at appreciable levels at the candidate off-target sites. For candidate off-target sites, the plotted values represent sequence modification frequencies relative to the genomic reference sequence, including substitutions, insertions, and deletions. For on-target sites, intended editing efficiencies are shown. Bars represent the mean, error bars represent SD, and dots represent individual values from three independent biological replicates.

We next compared N6RT with the parent RT editor at a panel of endogenous human genomic targets spanning substitutions, short insertions, and short deletions in HEK293T cells (Fig. 2a). Across these loci, intended-edit frequencies increased significantly with N6RT (paired t-test, *P* = 2.60 × 10^−8^). For example, at an EMX1 locus involving a 1-bp insertion, N6RT yielded 30.31 ± 1.39% intended edits versus 10.99 ± 3.50% with the RT-only control. Importantly, in all cases examined, indel frequencies remained low and showed no significant increase with the N6RT fusion compared to control (*P* = .28). We further stratified targets by edit class. In each class N6RT outperformed the RT control, with significant increases in intended editing for substitutions (*P* = 1.62 × 10⁻⁶), short insertions (*P* = .0092), and short deletions (*P* = .0057), as shown in Supplementary Fig. S1. These results establish the feasibility of PSBP augmentation for prime editing.

We next tested whether N6RT can improve editing at target-distal positions. Six distal edits were examined in EMX1 and UBE3A, covering substitutions, deletions, and insertions at positions +11, +16, and +21. N6RT increased intended editing efficiencies compared with RT at these tested distal edit sites (Supplementary Fig. S2). We further tested N6RT in two additional cell lines, HepG2 and HeLa. At three representative edit sites, N6RT increased intended editing efficiencies at most tested loci, although the extent of improvement varied by target site and cell type (Supplementary Fig. S3).

To examine whether the improved editing activity could be explained by increased protein accumulation, we compared the steady-state expression levels of HA-tagged RT and N6RT in HEK293T cells. N6RT did not show higher expression than RT by western blot analysis, suggesting that the enhanced editing efficiency is unlikely to result from increased steady-state protein abundance (Supplementary Fig. S4).

To further probe specificity, we analyzed candidate off-target loci for several representative targets, including EMX1, FANCF, HEK3, and HEK4. Products corresponding to the designed on-target intended edits were not detected at appreciable levels at these candidate off-target sites. We therefore quantified sequence modifications relative to the genomic reference sequence at each candidate off-target locus, including substitutions, insertions, and deletions. N6RT did not significantly increase these sequence modification frequencies compared with the RT control (Fig. 2c). These results indicate that N6RT improves on-target prime editing without causing a detectable increase in sequence modifications at the tested candidate off-target loci.

We also examined whether N6RT expression affected basic cell health in HEK293T cells. Compared with RT, N6RT did not cause obvious changes in cell morphology, cell proliferation measured by CCK-8 assay, or apoptosis measured by Annexin V-FITC/PI staining under the tested conditions (Supplementary Fig. S5).

### Compatibility of N6RT with PE3 nicking, MMR suppression, and engineered pegRNAs

**Figure 3.**
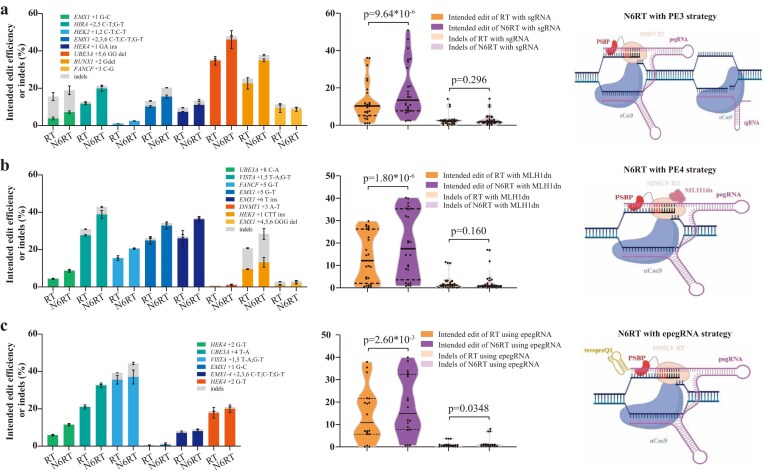
N6RT fusion editor is compatible with additional prime editing enhancements. (**a**) Editing outcomes at target loci using a PE3 strategy (addition of a secondary nicking sgRNA). The N6RT fusion yields higher intended edit percentages than the standard PE3 editor (RT-only), with minimal impact on indels (gray bars). (**b**) Prime editing outcomes under mismatch repair suppression (PE4 conditions, MLH1dn co-expression). N6RT further increases editing frequencies compared to the RT-only editor in the presence of MLH1dn, while indel rates remain low for both. (**c**) Editing efficiencies using structured pegRNA extensions (epegRNAs) with either the RT-only editor or N6RT. epegRNAs improve editing yields relative to standard pegRNAs, and the N6RT fusion provides an additional boost, achieving the highest observed editing frequencies.

We explored whether N6RT could be combined with other prime editing enhancements targeting different steps. We evaluated three such strategies in combination with N6RT: the addition of a nicking sgRNA on the non-edited strand (PE3 configuration) [1], transient MMR inhibition by MLH1dn co-expression (PE4 conditions) [4], and pegRNA 3′ extension stabilization using structured RNA elements (epegRNA) [8]. In all cases, N6RT provided an additional boost in editing efficiency on top of the baseline improvement, without increasing indel byproducts (Fig. 3).

For example, at a HIRA site, incorporating N6RT raised the PE3 editing frequency from 11.65 ± 0.50% to 19.89 ± 1.58% (Fig. 3a). Similarly, under MMR-inhibited conditions (PE4), N6RT further elevated editing yields beyond what MLH1dn alone achieved (Fig. 3b). When using epegRNAs, which protect the pegRNA 3′ tail, the N6RT fusion again drove higher editing efficiencies than the standard editor, for instance, increasing the editing percentage from 21.00 ± 1.16% to 32.55 ± 1.28% at a UBE3A site (Fig. 3c). Notably, indel rates remained low. Stacking these strategies can compound the gains in editing efficiency without appreciably increasing undesired byproducts. Together, these findings suggest that N6RT can be layered with existing PE improvements to achieve greater overall editing performance.

### N6RT synergizes with pegRNA tail binding, enables functional applications, and maintains activity in a single-component editor

**Figure 4.**
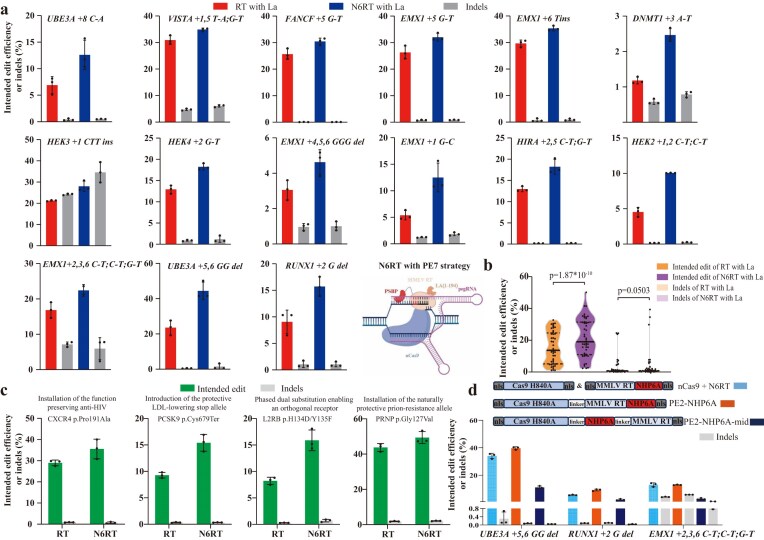
Synergistic enhancement by pegRNA tail-binding and one-component implementation. (**a**) Intended editing frequencies at 15 endogenous loci with the N6RT or RT-only editors with La domain. N6RT increases editing efficiency at all tested loci. (**b**) Indel frequencies corresponding to the conditions in panel (a). Indel rates remain low and comparable between RT + La and N6RT + La, indicating that higher editing does not come at the cost of specificity. (**c**) Installation of four example edits with clinical relevance (CXCR4 p.Pro191Ala, PCSK9 p.Cys679Ter, IL2RB p.His134Asp + p.Tyr135Phe, and PRNP p.Gly127Val) using the N6RT + La editor. All four alleles are edited efficiently with minimal indels. The N6RT + La system achieves higher editing frequencies than N6RT alone, with ~1.23×, 1.66×, 1.93×, and 1.13× increases at the respective loci. (**d**) Comparison of single component prime editors versus a two-component (split) N6RT editor at representative sites. The one-component PE2-NHP6A editor performs equivalently to the split system, maintaining similar editing efficiencies and low indel rates. Bars represent the mean, error bars represent SD, and dots represent individual values from three independent biological replicates.

A recent high-performance PE strategy, termed PE7 [11], was reported to dramatically improve editing by combining pegRNA tail stabilization and nicking. In PE7, the editor includes an La-assisted tail to stabilize pegRNA. We asked whether N6RT confers added benefit in the presence of La. We evaluated N6RT under La-assisted pegRNA tail stabilization and compared it directly with the parent RT editor under the same La background. Across all fifteen loci, N6RT with La produced higher intended edit frequencies than RT with La, while indel levels remained low and comparable (Fig. 4a and b). This shows that the N6RT-mediated enhancement persists in the presence of La.

We next applied this optimized PSBP+ La prime-editing system to install several functionally or clinically relevant edits in human cells. We chose four example targets that represent different editing scenarios: a missense mutation (CXCR4 p.Pro191Ala, relevant to WHIM syndrome), a nonsense (stop-gain) mutation (PCSK9 p.Cys679Ter, which reduces LDL cholesterol), a phased dual substitution (IL2RB p.His134As*P* + Tyr135Phe, two adjacent base changes in one edit window), and a protective polymorphism (PRNP p.Gly127Val, associated with prion-disease resistance). Using N6RT together with La, we achieved efficient editing at these sites, with fold changes of 1.23, 1.66, 1.93, and 1.13, respectively, and no significant indel increases (Fig. 4c).

Finally, we compared the performance of single-chain prime editor against the original split system. Encouragingly, the one-protein PE2-NHP6A editor showed comparable editing activity to the split design at all tested sites (Fig. 4d). Intended edit frequencies with single-chain PE2-NHP6A were not significantly different from those with split N6RT. Indel rates also remained similarly low. This indicates that PSBP augmentation can be consolidated into a single component prime editor without loss of efficiency, consistent with the finding that a split prime editor can function equivalently to an intact one.

## Discussion

PSBP fusion provides a minimalist and general strategy to boost prime editing efficiency while preserving precision. Mechanistically, a compact substrate-binding domain like NHP6A may help support the RT-associated substrate complex during reverse transcription, giving the RT more opportunity to complete DNA synthesis. This increases the fraction of successful edits without impeding other prime editing steps, as evidenced by the high product purity (low indels) and no detectable increase in candidate off-target sequence modifications observed with N6RT.

Our results show that PSBP augmentation is highly complementary to other prime editing enhancements. Because N6RT fortifies the extension step, it can be combined with strategies addressing different bottlenecks such as strand nicking (PE3), mismatch repair suppression (PE4), pegRNA extension stabilization (epegRNA), or tail-binding domains (PE7). We found that stacking these improvements yielded multiplicative gains in editing efficiency without sacrificing fidelity. Nevertheless, the magnitude of improvement by N6RT varied among target sites, which is consistent with the context-dependent nature of prime editing. Editing outcomes can be influenced by local sequence context, edit position, PBS and RTT design, pegRNA structure, chromatin state, and cellular context. Thus, N6RT should be considered a modular strategy to improve the RT extension step, rather than a universal solution for all low-efficiency targets. Its compatibility with PE3, PE4, epegRNAs, and La-assisted pegRNA stabilization suggests that N6RT can be combined with other strategies when higher editing efficiency is needed.

Previous studies using DNA-binding domains have shown that strengthening the interaction between prime editors and nucleic acid substrates can improve editing efficiency [26]. Our work further expands this strategy by screening polymerase substrate-binding proteins from bacterial, archaeal, and eukaryotic sources. The finding that most tested PSBP fusions increased editing efficiency suggests that polymerase-associated substrate-binding proteins provide a useful source of compact functional modules for prime editor engineering. These results also indicate that additional natural or engineered substrate-binding factors can be explored to further improve prime editing performance.

The practical benefit of PSBP-augmented prime editors is illustrated by the efficient installation of disease-relevant gene edits with minimal byproducts. For example, our N6RT plus La system precisely introduced the protective PRNP p.G127V variant in >50% of alleles with ~98% product purity. Similarly, we achieved high editing efficiency at other clinically relevant loci (such as CXCR4 and PCSK9), underscoring the potential of these enhancements for research and therapeutic applications. Further optimization in delivery and cell-specific contexts may extend these gains to more cell types. A practical consideration for PSBP-enhanced prime editors is cargo size. Although NHP6A is a compact protein of ~93 amino acids, its fusion still increases the size of the already large prime editor. For AAV-based delivery, full-length prime editors are already difficult to package into a single AAV vector and generally require split or dual-AAV strategies. In this context, the additional size contributed by NHP6A is relatively small and may be compatible with split-AAV delivery designs. Future applications of N6RT-containing editors may also benefit from alternative delivery strategies, such as LNP-mediated mRNA delivery.

More broadly, our work demonstrates that recruiting natural polymerase accessory factors can significantly improve a genome editing enzyme. In nature, replicative DNA polymerases achieve high processivity by tethering to ring-shaped sliding clamps (PCNA in eukaryotes, β clamp in bacteria). Phage and virus DNA polymerases, although mostly coming alone due to their compact genomes, similarly often require host proteins for processivity. For instance, T7 DNA polymerase binds the *E. coli* thioredoxin to remain stably associated with DNA [27]. RNA polymerases use elongation factors like Spt4/5 (NusG in bacteria) to clasp the transcription complex and enable continuous RNA synthesis through impediments [28]. Even specialized reverse transcriptases exploit host factors: telomerase, a cellular RT, requires the TPP1–POT1 complex to achieve processive DNA repeat addition [29], and the hepatitis B virus RT must recruit the host chaperone Hsp90 to initiate cDNA synthesis [30]. These examples illustrate a general principle that polymerases often operate in concert with accessory proteins that secure the enzyme-substrate complex. By adopting this principle, fusing a small duplex-stabilizing module to the prime editor’s RT, we effectively bolstered the editor’s grip on the primer-template, converting more editing attempts into successful outcomes. Notably, just as fusing duplex-binding proteins (like Sso7d) has enhanced DNA polymerases for PCR [13], fusing an HMG-box PSBP to the prime editor’s RT fortifies its activity on the RNA-DNA hybrid. In this work, we performed a screen of 12 PSBPs in N- and C-terminal RT-fusion orientations and identified multiple active fusions, including N6RT, highlighting the potential of exploring natural and engineered PSBPs to improve PE performance. This translates a well-established principle from natural polymerase assemblies to a genome-editing context and opens a new class of editor upgrades based on compact accessory modules. The strategy is minimal, portable, and compatible with existing toolkits, making it immediately useful for groups seeking higher prime-editing yields across edit types and loci.

In conclusion, PSBP fusion offers a straightforward and precise route to improve prime editing outcomes. Adding a small substrate-binding domain to the editor does not require additional guide RNAs or small molecules, yet yields substantial efficiency gains and works synergistically with other enhancements. This strategy can be readily integrated into next-generation prime editors alongside evolved RT enzymes and streamlined Cas9 architectures. As genome editing moves toward therapeutic uses, combining orthogonal improvements will be crucial to achieve the necessary levels of efficiency and fidelity. By adopting a principle long used by nature’s polymerases, our prime editor with NHP6A provides a powerful component in the genome editing toolbox.

## Supplementary Material

gkag657_Supplemental_File

## Data Availability

There is no restriction on data associated with this study. High-throughput sequencing data have been deposited in NCBI Sequence Read Archive (SRA) under accession code PRJNA1475255. This study relied on publicly available software (CRISPResso2) [15] for data processing with no restrictions on code usage.
