# Supplementary material for: Enhancing prime editing by fusing polymerase substrate-binding proteins to reverse transcriptase
Source: Nucleic Acids Res. 2026 Jun 27;54(12):gkag657. doi: 10.1093/nar/gkag657 (PMC13309780; doi:10.1093/nar/gkag657)
Supplement: gkag657_Supplemental_File [file gkag657_supplemental_file.pdf]

Supplementary Information

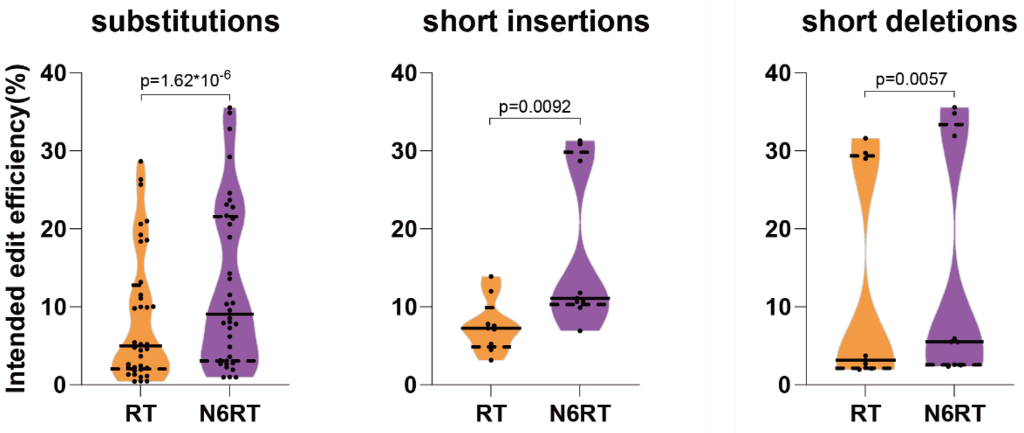

Supplementary Figure 1. N6RT increases intended editing across edit classes. Intended edit efficiencies for substitutions, short insertions, and short deletions in HEK293T cells are shown for the RT control and the N6RT fusion. Each dot represents one biological replicate at a tested locus. Violin shapes depict the distribution across loci. Horizontal bars indicate the median. Paired t tests compare N6RT with RT within each edit class, with p values reported above the plots.

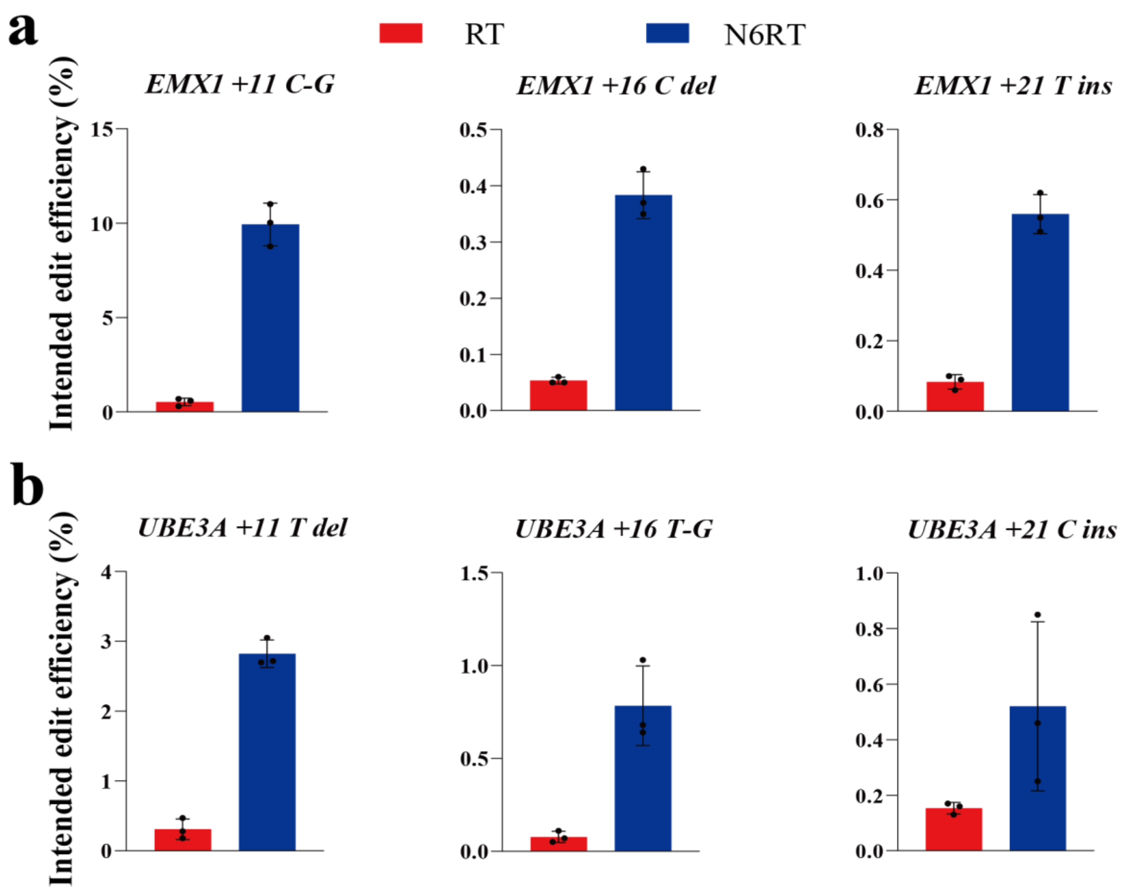

Supplementary Figure 2. N6RT improves editing efficiencies at target-distal edit sites. Editing outcomes at distal edit sites in HEK293T cells comparing the RT-only editor with the N6RT fusion editor. **a.** EMX1 distal edits. **b.** UBE3A distal edits. Data are presented as mean ± SD from three independent experiments.

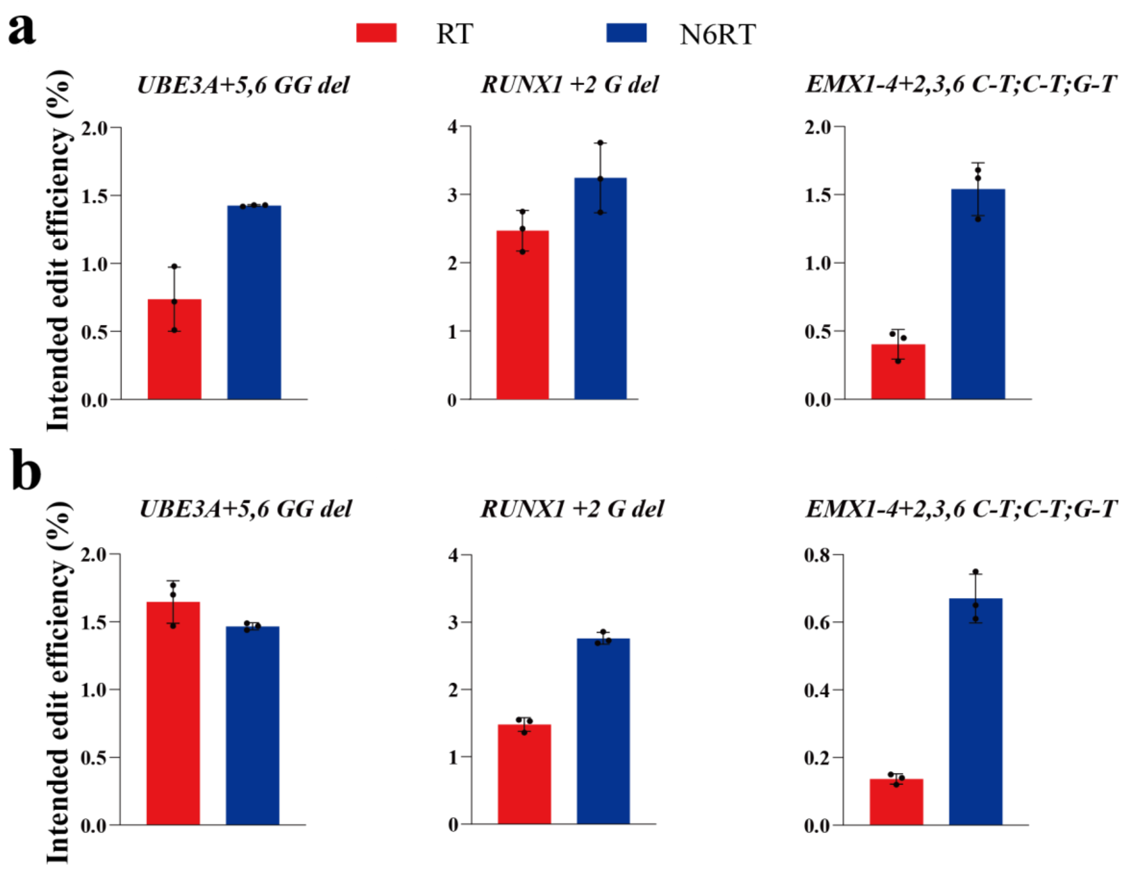

Supplementary Figure 3. N6RT fusion editor in HepG2 and HeLa cells. Editing outcomes at representative endogenous loci in HepG2 and HeLa cells comparing the RT-only editor (red bars) with the N6RT fusion editor (blue bars). **a.** HepG2 cells. **b.** HeLa cells. Data are presented as mean ± SD from three independent experiments.

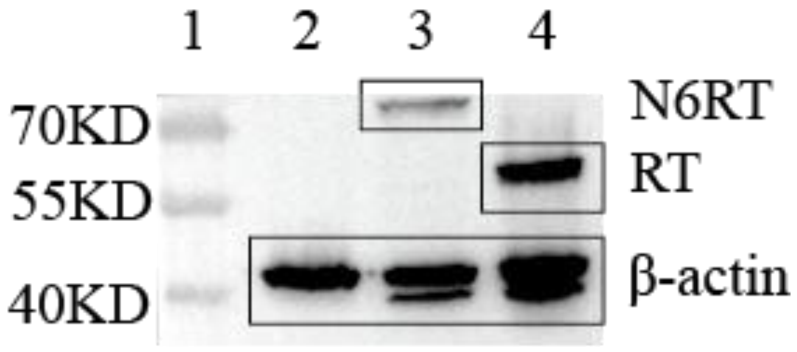

Supplementary Figure 4. Expression levels of RT and N6RT proteins in HEK293T cells. Whole-cell lysates were collected 24 h after transfection for western blot analysis using anti-HA and anti-β-actin antibodies. Lane 1, marker; lane 2, negative control cells transfected without RT or N6RT expression plasmid; lane 3, cells transfected with N6RT; lane 4, cells transfected with RT.

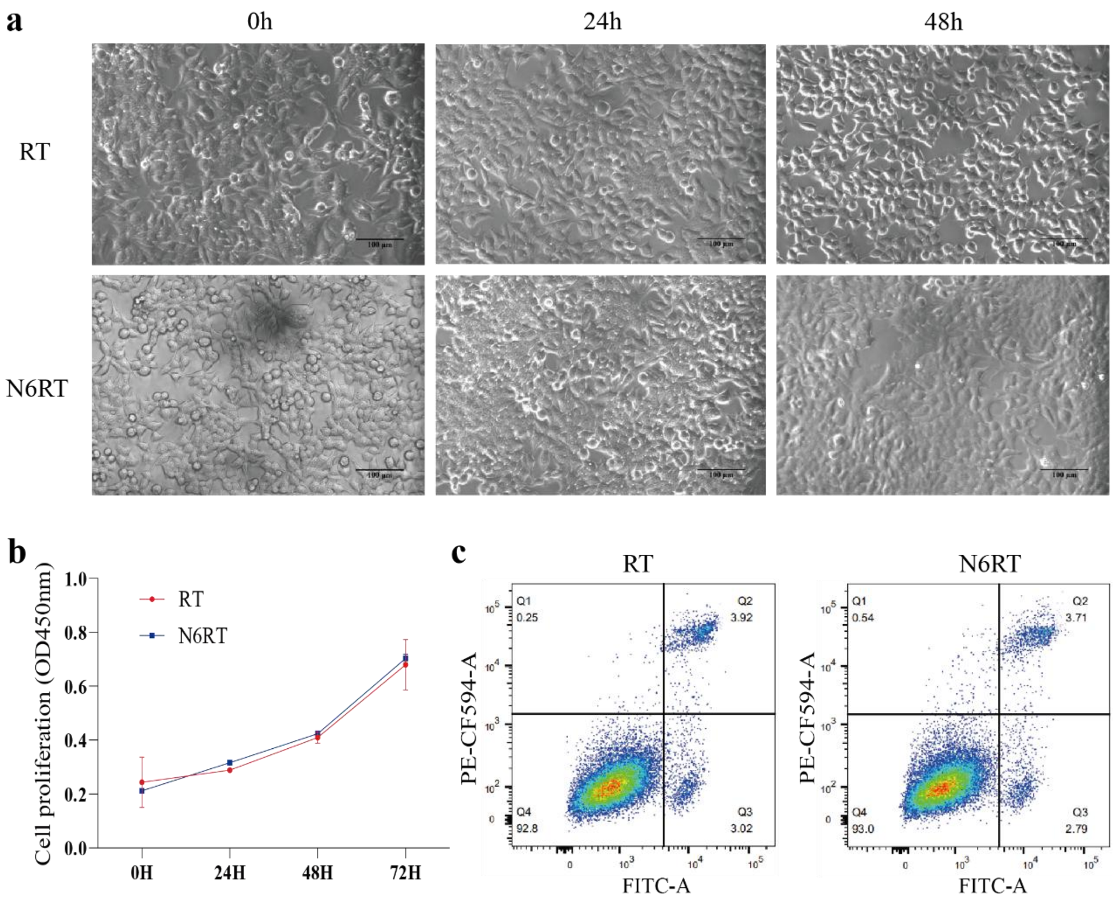

Supplementary Figure 5. Comparison of cellular morphology, proliferation, and apoptosis between RT- and N6RT-transfected HEK293T cells. a. Representative bright-field images of HEK293T cells transfected with RT or N6RT at the indicated time points. Scale bars, 100 μm. **b.** Cell proliferation was quantified using the CCK-8 assay over a 72 h period. Data are presented as mean ± SD from three independent experiments. **c.** Apoptosis was analyzed by flow cytometry using Annexin V-FITC/PI staining. Representative flow cytometry plots are shown, and the percentages of cells in each quadrant are indicated.

Supplementary Table 1. Protospacer and 3′ extension sequences of pegRNAs/epegRNAs and nicking sgRNA protospacers

Target sites	pegRNA protospacer	RT	PBS	Nicking gRNA protospacer
EMX1 +5 G-T	GAGTCCGAGCAG AAGAAGAA	ATGGGAGCAC TTC	TTCTTCTGCTC GGAC (15nt)	
EMX1 +6 T ins	GAGTCCGAGCAG AAGAAGAA	ATGGGAGCAC CTTC	TTCTTCTGCTC GGAC (15nt)	
EMX1 +4,5,6 GGG del	GAGTCCGAGCAG AAGAAGAA	ATGGGAGTTC	TTCTTCTGCTC GGAC (15nt)	
EMX1 +1 G-C	GAGTCCGAGCAG AAGAAGAA	GTGATGGGAG CCCTTG	TTCTTCTGCTC GG (13nt)	GCCGTTTGTAC TTTGTCCTC
EMX1 +2,3,6 C-T;C-T;G-T	GTATTCATTTCCC TAGCTCC	TAGCCGTCACT AAA	GCTAGGGAAA TGAATA (16nt)	GTCCCTTTCCA CTTTGCTAA
FANCF +5 G-T	GGAATCCCTTCTG CAGCACC	GGAAAAGCGA TCAAGGT	GCTGCAGAAG GGATT (15nt)	
FANCF +3 C-G	GGAATCCCTTCTG CAGCACC	GGAAAAGCGA TCCACGT	GCTGCAGAAG GGAT (14nt)	GGGGTCCCAG GTGCTGACGT
UBE3A +8 C-A	GTACAGTTAGTAC TCAGCAG	TTTGAGATTCC ACTG	CTGAGTACTA ACT (13nt)	
UBE3A +4 T-A	GTACAGTTAGTAC TCAGCAG	TGAGAGTCCTC TG	CTGAGTACTA ACT (13nt)	
UBE3A +5,6 GG del	GTACAGTTAGTAC TCAGCAG	TTTGAGAGTAC TG	CTGAGTACTA ACT (13nt)	TGTCTGAGAA GGCAGAGAA
HEK4 +1 GA ins	GGCACTGCGGCTG GAGGTGG	TTAACCCCCTC A	CCTCCAGCCG C (11nt)	AGACACACAC ACAGGCCTGG
HEK4 +2 G-T	GGCACTGCGGCTG GAGGTGG	TTAACCCCAA	CCTCCAGCCG C (11nt)	
VISTA +1,5 T-A;G-T	GAACACAAAGCA TAGACTGC	GGCCCGCCAC GCT	GTCTATGCTT TGTGT (15nt)	
DNMT1 +3 A-T	GATTCCTGGTGCC AGAAACA	CCCGTCACCCC AGT	TTCTGGCACC AGG (13nt)	
HIRA +2,5 C-T;G-T	GTATTCTAGAATG CAGGGCA	CCTGTTTCCAC TAC	CCTGCATTCT AGAAT (15nt)	GTGAAGTGCC CTTCCTCCTT
HEK2 +1,2 C-T;C-T	GAATACTTCTTCA GAGTCCC	GAGTATCCTG AA	ACTCTGAAGA AGTAT (15nt)	CCCACACCATT TTTTAAAAA
HEK3 +1 CTT ins	GGCCCAGACTGA GCACGTGA	TCTGCCATCAA AG	CGTGCTCAGT CTG (13nt)	
RUNX1 +2 G del	GCATTTTCAGGAG GAAGCGA	TGTCTGAAGCC ATG	CTTCCTCCTG AAAAT (15nt)	ATGAAGCACT GTGGGTACGA
VEGFA+1 T-A	GATGTCTGCAGGC CAGATGA	AATGTGCCATC TGGAGCCCTCT	TCTGGCCTGC AGA (13nt)	

PRNP +6 G-T	GCAGTGGTGGGG GGCCTTGG	ATGTAGACGC CA	AGGCCCCCCA CC (12nt)
CXCR4 +5 G-C	GCAACCACCCACA AGTCATTG	TGACCGCTTCT ACGCCAA	TGACTTGTGG GTGGT (15nt)
IL2RB +1,5 T-A;G-C	GCCAGGTGTCTTT CAAAGTAG	TCCCAAGCCTC CGACTT	CTTTGAAAGA CAC (13nt)
PCSK9 +3 C-A	GCAGCCGTTGCCA TCTGCTGC	GCCGGCTCCGT CA	GCAGATGGCA ACG (13nt)
EMX1 +11 C-G	GAGTCCGAGCAG AAGAAGAA	GGTTGATGTG ATCGGAGCCC TTC	TTCTTCTGCTC GGAC (15nt)
EMX1 +16 C del	GAGTCCGAGCAG AAGAAGAA	CCACCGGTTG ATTGATGGGA GCCCTTC	TTCTTCTGCTC GGAC (15nt)
EMX1 +21 T ins	GAGTCCGAGCAG AAGAAGAA	TGCGCCACCG GTATGATGTG ATGGGAGCCC TTC	TTCTTCTGCTC GGAC (15nt)
UBE3A +11 T del	GTACAGTTAGTAC TCAGCAG	ATCTGCCATTT GGAGTCCACT G	CTGAGTACTA ACT (13nt)
UBE3A +16 T-G	GTACAGTTAGTAC TCAGCAG	CAGAAATCTG CCCTTTGAGAG TCCACTG	CTGAGTACTA ACT (13nt)
UBE3A +21 C ins	GTACAGTTAGTAC TCAGCAG	GCTTCAGAAA TCGTGCCATTT GAGAGTCCAC TG	CTGAGTACTA ACT (13nt)

Supplementary Table 2. The original and intended sequences for each locus edited*

Target sites	Original genomic sequence	Intended edited sequence
EMX1 +5 G-T	GAAGGGCTCCCATCACATCA	GAAGTGCTCCCATCACATCA
EMX1 +6 T ins	GAAGGGCTCCCATCACATCA	GAAGGTGCTCCCATCACATCA
EMX1 +4,5,6 GGG del	GAAGGGCTCCCATCACATCA	GAACTCCCATCACATCA
EMX1 +1 G-C	GAAGGGCTCCCATCACATCA	CAAGGGCTCCCATCACATCA
EMX1 +2,3,6 C-T;C-T;G-T	TCCAGGGACGGCTATACCAG	TTTAGTGACGGCTATACCAG
FANCF +5 G-T	ACCTGGATCGCTTTTCCGAG	ACCTTGATCGCTTTTCCGAG
FANCF +3 C-G	ACCTGGATCGCTTTTCCGAG	ACGTGGATCGCTTTTCCGAG
UBE3A +8 C-A	CAGTGGACTCTCAAATGGCA	CAGTGGAATCTCAAATGGCA
UBE3A +4 T-A	CAGTGGACTCTCAAATGGCA	CAGAGGACTCTCAAATGGCA
UBE3A +5,6 GG del	CAGTGGACTCTCAAATGGCA	CAGTACTCTCAAATGGCA
HEK4 +1 GA ins	TGGGGGTTAAAGCGGAGACT	GATGGGGGTTAAAGCGGAGACT
HEK4 +2 G-T	TGGGGGTTAAAGCGGAGACT	TTGGGGTTAAAGCGGAGACT

VISTA +1,5 T-A;G-T	TGCGGGGCGGGCCAGCCTGA	AGCGTGGCGGGCCAGCCTGA
DNMT1 +3 A-T	ACAGGGGTGACGGGAGGGCA	ACTGGGGTGACGGGAGGGCA
HIRA +2,5 C-T;G-T	GCAGGGGAAACAGGAGCTAG	GTAGTGGAAACAGGAGCTAG
HEK2 +1,2 C-T;C-T	CCCAGGATACTCTTCAAAGT	TTCAGGATACTCTTCAAAGT
HEK3 +1 CTT ins	TGATGGCAGAGGAAAGGAAG	CTTTGATGGCAGAGGAAAGGAAG
RUNX1 +2 G del	CGATGGCTTCAGACAGCATA	CATGGCTTCAGACAGCATA
VEGFA+1 T-A	TGAGGGCTCCAGATGGCACA	AGAGGGCTCCAGATGGCACA
PRNP +6 G-T	TGGCGGCTACATGCTGGGAA	TGGCGTCTACATGCTGGGAA
CXCR4 +5 G-C	TTGGGGTAGAAGCGGTCACA	TTGGCGTAGAAGCGGTCACA
IL2RB +1,5 T-A;G-C	TAGTGGGAGGCTTGGGAGAT	AAGTCGGAGGCTTGGGAGAT
PCSK9 +3 C-A	TGCCGGAGCCGGCACCTGGC	TGACGGAGCCGGCACCTGGC
EMX1 +11 C-G	GAAGGGCTCCCATCACATCAACCGG	GAAGGGCTCCGATCACATCAACCGG
EMX1 +16 C del	GAAGGGCTCCCATCACATCAACCGG	GAAGGGCTCCCATCAATCAACCGG
EMX1 +21 T ins	GAAGGGCTCCCATCACATCAACCGG	GAAGGGCTCCCATCACATCATACCGG
UBE3A +11 T del	CAGTGGACTCTCAAATGGCAGATTT	CAGTGGACTCCAAATGGCAGATTT
UBE3A +16 T-G	CAGTGGACTCTCAAATGGCAGATTT	CAGTGGACTCTCAAAGGGCAGATTT
UBE3A +21 C ins	CAGTGGACTCTCAAATGGCAGATTT	CAGTGGACTCTCAAATGGCACGATTT

*Nucleotide positions are numbered relative to the Cas9 nick site, with the first nucleotide after the nick defined as position +1.

Supplementary Table 3. Deep sequencing primers

Target sites	Oligo-F	Oligo-R
EMX1 +5 G-T	GACAAAGTACAAACGGCAGA	AGCAGCACTCTGCCCTCGT
EMX1 +6 T ins	GACAAAGTACAAACGGCAGA	AGCAGCACTCTGCCCTCGT
EMX1 +4,5,6 GGG del	GACAAAGTACAAACGGCAGA	AGCAGCACTCTGCCCTCGT
EMX1 +1 G-C	GACAAAGTACAAACGGCAGA	AGCAGCACTCTGCCCTCGT
EMX1 +2,3,6 C-T;C-T;G-T	GCTCCCTCATTCCTGGGAATCT	GGGCTGTCAGTTTCGGACTAGG
FANCF +5 G-T	AGTCGCCGTCTCCAAGGTGAAA	CGATGGATGTGGCGCAGGTA
FANCF +3 C-G	AGTCGCCGTCTCCAAGGTGAAA	CGATGGATGTGGCGCAGGTA
UBE3A +8 C-A	CAGCTTACTGCCCCTCTTTC	TCAGAATCAAGAGATCAGAGCTGAG
UBE3A +4 T-A	CAGCTTACTGCCCCTCTTTC	TCAGAATCAAGAGATCAGAGCTGAG
UBE3A +5,6 GG del	CAGCTTACTGCCCCTCTTTC	TCAGAATCAAGAGATCAGAGCTGAG

HEK4 +1 GA ins	GGCGAGGCAGAGGGTCCAAA	TTTCCCGGGCTCCTTTCAACC
HEK4 +2 G-T	GGCGAGGCAGAGGGTCCAAA	TTTCCCGGGCTCCTTTCAACC
VISTA +1,5 T-A;G-T	GGCAGGACGTCTGCCCAATA	CCCCATCTGTCAAACTGTGCG
DNMT1 +3 A-T	CCACACATGTGAACGGACAG	TGTCAAGTGGCGTGACACCG
HIRA +2,5 C-T;G-T	GGTAAGAGTTCTGTGGGGACCAG	AGGTTCAGTCCAAGGACACATCC
HEK2 +1,2 C-T;C-T	GAAGGACATCTGGGAGTGCAGT	GAGTTAAGCGGGGCCATAAGAT
HEK3 +1 CTT ins	TTTTTCTGCTTCTCCAGCCC	TTCCAGCCCAGCCAAACTTG
RUNX1 +2 G del	CGCTCCGAAGGTAAAAGAAATC	ACAAGCTGCCATTTCATTACAG
VEGFA+1 T-A	TTCCCAAAGGACCCCAGTCA	GACCTCCCAAACAGCTACAT
PRNP +6 G-T	CACAGTCAGTGGAACAAGCCG	GCTGTACTCATCCATGGGCCT
CXCR4 +5 G-C	TTGAGGGCCTTGCGCTTCTG	CCTGCCCTCCTGCTGACTATT
IL2RB +1,5 T-A;G-C	GGGACAGGACATGGACCAGG	TCTCCAGTTCGCCTGATGGCC
PCSK9 +3 C-A	CCTACGCCGTAGACAACACG	GCCATGGAGGGCTGAGAGAG
EMX1 +11 C-G	GACAAAGTACAAACGGCAGA	AGCAGCACTCTGCCCTCGT
EMX1 +16 C del	GACAAAGTACAAACGGCAGA	AGCAGCACTCTGCCCTCGT
EMX1 +21 T ins	GACAAAGTACAAACGGCAGA	AGCAGCACTCTGCCCTCGT
UBE3A +11 T del	CAGCTTACTGCCCCTCTTTC	TCAGAATCAAGAGATCAGAGCTGAG
UBE3A +16 T-G	CAGCTTACTGCCCCTCTTTC	TCAGAATCAAGAGATCAGAGCTGAG
UBE3A +21 C ins	CAGCTTACTGCCCCTCTTTC	TCAGAATCAAGAGATCAGAGCTGAG

Supplementary Sequences 1. Amino acid sequences of PSBPs.

BS:

MSSKIKEFSKEGIGSVKTIDEITQFRGNEERYAIIAEAKSPGVGSIGQLEE

COMEA:

MGEETAVQQGGGGSVQSDGGKGALVNINTATLEELQGISGVGPSKAEAIIAYR

EENGRFQTIEDITKVSGIGEKSFEKIKSSITVK

NHP6A:

MVTPREPKKRTTRKKKDPNAPKRALSAYMFFANENRDIVRSENPDITFGQVG

KKLGEKWKALTPEEKQPYEAKAQADKKRYESEKELYNATLA

SSO7D2:

MMATVKFKYKGEEKEVDISKIKKVWRVGKMISFTYDEGGGKTGRGAVSEKD

APKELLQMLEKQKK

SSO7D:

MKKQKELMQLLEKPKDKESVAGRGTKGGGEDYTFSIMKGVRWVKKIKSIDV

EKEEGKYKFKVTA

T3TBD:

MGSWYQPKGGTEMFCHPRTGKPLPKYPRIKIPKVGGIFKKPKNKAQREGREP

CELDTREYVAGAPYTPVEHVVFNPS

T7TBD:

MGSWYQPKGGTEMFCHPRTGKPLPKYPRIKTPKVGGIFKKPKNKAQREGREP

CELDTREYVAGAPYTPVEHVVFNPS

TA:

MGNTLHDAVVAEIADQEAGCIDLNRASVSELTALPHIGEARAEAIKDGRPWNA

VRDLKEIRGIGAGRLEEIKARGEACIEP

TD01:

MSNKITEVSAVGLGSVQSLEDANTWPRMEIIQEARVPGVNPLETLQEATAENL

DICYIPDNA

TK:

MRAKIEELRGAGIGRIEKLDRVANWPRGDKIAEARAEGIHPLATLES

TOPV:

MARERVESRRKSGRQERSEEEWKEWLERKVGEGRARRLIEYFGSAGEVGKL
VENAEVSKLLEVPGIGDEAVARLVPGYKTLRDAGLTPAEAERVLKRYGSVSKV
QEGATPDELRELGLGDAKIARILGLRSLVNKRLDVDTAYELKRRYGSVSAVRK
APVKELRELGLSDRKIARIKGIPETMLQVRGMSVEKAERLLERFDTWTKVKE
APVSELVRVPGVGLSLVKEIKAQVDPAWKALLDVKGVSPELADRLVEELGSPY
RVLTAKKSDLMRVERVGPKLAERIRAAGKRYVEERRSRRERIRRKLRG

TthRecA:

MDESKRKALENALKAIEKEFGKGAVMRLGEMPKQQVDVIPTGSLALDLALGI
GGIPRGRIVEIYGPESGGKTTLALTIIAQAQRRGGVAAFVDAEHALDPLYAQRL
GVQVEDLLVSQPDTGEQALEIVELLARSGAVDVIVVDSVAALVPRAEIEGEMG
DQHVGLQARLMSQALRKLTAVLAKSNTAAIFINQVREKVGVTYGNPETTPGG
RALKFYASVRLDVRKSGQPIKVGNEAVGVKVRVKVVKNKLAPPFREAELEIY
FGRGLDPVADLVNVAVAAGVIEKAGSWFSYGELRLGQGKEKAAEALRERPEL
LEEIRAKVLERSDQVVLAAGEDEGEGSGGSG
